# The relationship between risk factors for falling and the quality of life in older adults

**DOI:** 10.1186/1471-2458-5-90

**Published:** 2005-08-26

**Authors:** Ayse Ozcan, Hulya Donat, Nihal Gelecek, Mehtap Ozdirenc, Didem Karadibak

**Affiliations:** 1 Dokuz Eylül University School of Physical Therapy and Rehabilitation, 35340 Inciralti-Izmir, Turkey; 2 T.C. Emekli Sandigi Narlidere Nursing Home (The Izmir Geriatric Centre), 35340 Narlidere-Izmir, Turkey

## Abstract

**Background:**

Falls are one of the major health problems that effect the quality of life among older adults. The aim of this study was to explore the relationship between quality of life (Short Form-12) and the risk factors of falls (balance, functional mobility, proprioception, muscle strength, flexibility and fear of falling) in older adults.

**Methods:**

One hundred sixteen people aged 65 or older and living in the T.C. Emekli Sandigi Narlidere nursing home participated in the study. Balance (Berg Balance test), functional mobility (Timed Up and Go), proprioception (joint position sense), muscle strength (back/leg dynamometer), flexibility (sit and reach) and fear of falling (Visual Analogue Scale) were assessed as risk factors for falls. The quality of life was measured by Short Form-12 (SF-12).

**Results:**

A strong positive correlation was observed between Physical Health Component Summary of SF-12, General Health Perception and balance, muscle strength. Proprioception and flexibility did not correlated with SF-12 (p > 0.05). There was negative correlation between Physical Health Component Summary of SF-12, General Health Perception and fear of falling, functional mobility (p < 0.05).

**Conclusion:**

We concluded that the risk factors for falls (balance, functional mobility, muscle strength, fear of falling) in older adults are associated with quality of life while flexibility and proprioception are not.

## Background

Quality of life is a term used in a number of disciplines, and definitions and conceptualization varies from utility of health states to life satisfaction, and from possession of socially desirable characteristics to positive affect [1]. Quality of life has recently become commonly used both as a concept and as a field of research [2-5]. Studies have also demonstrated that older elderly people expressed higher life satisfaction/quality of life than younger ones.

There are many socio-demographic characteristics that may contribute to the quality of life such as age, socio-economic status, and marital status in older adults. Falls are one of the major health problems that effect the quality of life among older adults [2-5]. The Short-Form 36 (SF-36) is a widely used quality of life instrument. However, its length could affect response rates, particularly in older adults. The SF-12 has proved to be suitable for older adults because of the limited number of questions. The 12 items in SF-12 represent one physical component summary score and one mental component summary score and assess a person's perceived health-related quality of life.

Many factors were originally considered as possible risk factors for falls based on a review of currently available literature. These factors include age, number of chronic diseases, body composition, muscle strength, functional mobility and performance measures related to balance function [3,4,6].

Impaired balance and functional mobility are major risk factors for falls. There are many studies investigating the relationship between falling and contributory factors [7-13]. However, no study investigating the correlation between risk factors for falls and quality of life in older adults could be found.

Since falls and its consequences have a major role in quality of life, rehabilitation programs, which aim to decrease the risk of falling by considering all contributing factors such as muscle strength, flexibility and balance, have the potential to both decrease the risk of falling and improve the quality of life. Due to this interaction, the relationship between risk factors for falls and the quality of life becomes significant. Based on a review of literature, this study was designed to explore the relationship between the quality of life (Short Form-12) and risk factors for falls (balance, functional mobility, proprioception, muscle strength, flexibility and fear of falling) in older adults.

## Methods

### Subjects

A total of 116 (52 men and 64 women) participants aged 65 or older with or without a history of falls were recruited from the 535 registered residents of T.C. Emekli Sandigi Narlidere nursing home for this study. Ambulatory individuals having no disability in self-care formed the population of this study and a report stating sound mental healthy from a psychiatrist of a state hospital was required at the registration of all participants.

The exclusion criteria were as follows: being aged less than 65, being unable to walk less than 10 meters, amputation, having had a stroke recently, unstable medical conditions such as diabetes mellitus, hypertension, 2 or more fractures due to osteoporosis, resting angina, recurrent heart failure or recurrent arrhythmias and uncontrolled seizure disorder. Also the residents who were assessed as mentally oriented by the psychiatrist were included the study. After checking health documents of residents, and considering the inclusion criteria, 404 residents were approached about the study. 112 subjects did not agree to participate, and 53 subjects exercised regularly (more than twice a week during the previous 2 months). 37 potential subjects couldn't be reached. 202 subjects accepted the invitation to participate in the study and 141 of them came and were evaluated. The evaluation of 25 subjects couldn't be completed the assessment because they were not able to take some positions of tests physically. Data were obtained from 116 subjects.

The study was performed according to the principles of the Decleration of Helsinki and was approved by the Ethics Committee of Dokuz Eylül University Medical School (reference number: 29.12.03/156). Informed consent was taken from the patients, immediately prior to the data collection. After giving informed consent, all subjects completed a health status questionnaire which provided information on age, medical history, alcohol consumption, self-reported history of fall, use of devices to assist ambulation and medication. The same physiotherapist did all assessments.

### Procedure

#### The quality of life

The quality of life was measured by Short Form-12 (SF-12) (Ware, Kosinski and Keller 1996). The items in the SF-12 instrument were used to calculate two scales, the Physical Component Scale and the Mental Component Scale. Scores range from 0 to 100, a higher score indicates better mental health, physical health and general health perception [14].

#### Balance

The Berg Balance Scale (BBS) was used to evaluate balance. The BBS is a 14-item balance assessment tool that is scored on a 5 point ordinal scale (0–4) measuring levels of ability in performing each task (4 = safe and independent, 0 = incapable). The BBS includes tasks such as standing with eyes closed, reaching, standing on one foot and picking up objects from the floor. The highest total possible score on the Berg Balance Scale is 56, indicating excellent balance [15].

#### Functional mobility

The Timed Up and Go (TUG) test was used to measure basic functional mobility. The time taken to complete rising from a chair, walking 10 ft (3 m), turning, walking back to the chair and sitting was recorded in seconds. The starting position was standardized so that the subjects commenced the test with their feet flat on the floor and their arm resting on the armrests. No physical assistance was given. Each subjects was asked to perform three test trials. The mean score was recorded [16].

#### Proprioception (joint position sense)

Proprioception was assessed using established and validated lower limb matching tasks. In this test, subjects seated with their eyes closed were asked to align their lower limbs simultaneously on either side of a vertical clear acrylic sheet (60 × 60 × 1 cm) inscribed with a protractor and placed between their legs. To prevent limited motion at the knee joint from confounding the results of this test, the examiner needed to ensure that subjects matched their limbs near the midrange of knee joint motion. Each trail was undertaken relatively quickly, with rests between trials, to avoid weakness unduly influencing the results. Any difference in aligning the lower limbs (indicated by disparities in matching the big toes on either side of acrylic sheet) was measured in degrees for both extremities. After 2 practice trails, an average of 5 experimental trails was recorded [17].

#### Muscle strength

Back/leg dynamometer was used to measure leg strength. The subject stood on a platform with their feet apart at a comfortable distance of shoulder width for balance. Their hands grasped each end of a bar. The subject was asked to flex at their knees to approximately 135 degrees. The back was kept straight and the hips were positioned directly over the ankle joints. In this way, the activation of back muscles was eliminated. The chest was kept forward and the head was held in an erected position. The subject took in a large breath and slowly exhaled as they attempted to extend their knees smoothly and as forcefully as possible. Three attempts were made and a mean score was recorded [18].

#### Flexibility

In order to assess flexibility, a sit and reach test was used. A box 32 cm in height and 50 cm in length with a top plate 45 cm in width was used for the test. The length of the top plate was 75 cm, the first 25 cm of which was extended over the front edge of the box towards the subject's feet. Older adults were asked to sit, keeping their knees straight, and reach forward as far as possible from a seated position. The score was determined by the furthest position they reached with their fingertips on a scale. Three trials were perfomed and the mean score was recorded [19,20].

#### Fear of falling

As an indication of fear of falling in daily life a visual analogue scale (VAS) was used. Subjects were asked to express their overall feelings of fear of falling by drawing a mark on a vertical line of exactly 10 centimetres connecting the two statements: "no fear of falling" (below) and "very afraid of falling"(above). The score was the number of centimetres between "no fear of falling" and the subject's mark [21].

### Statistical analysis

Applying SPSS version 10.0 for statistical analyses, we considered differences of two-tailed p < 0.05 as statistically significant. All data were shown as means with standard deviations (means ± SD) and ranges were added. Pearson's correlation coefficient was used to analyse the relationship between the SF-12 and balance, functional mobility, proprioception, muscle strength, flexibility and fear of falling.

## Results

Demographic characteristics of the subjects and medical history were summarized in Table 1.

**Table 1:** Demographic characteristics of subjects (n = 116)

		n	%
Age (X ± SD) (Range) (years)	76.60 ± 6.19 (65–90)		
BMI (X ± SD) (Range)(kg/m^2^)	27.69 ± 3.46 (20.9–40.0)		
Sex (n)	Female	64	55
	Male	52	45
Assistive device	No	96	82
	Yes	20	17
Alcohol consumption	No	94	81
	Yes	22	19
Fall(s) in the previous year	No	79	68
	1 time	25	22
	2 times or more	12	10
Reported medical conditions	Osteoarthritis	47	
	Hypertension	41	
	Osteoporosis	34	
	Low back pain	21	
	Visual and hearing problems	20	
	Cardiac problems	14	
	Urinary incontinence	7	
	Peripheral vascular disease	4	
	Asthma	4	
No of medications (X ± SD) (Range)	4.03 ± 2.42 (0–10)		

Means ± SD and range of all measurements performed are given in Table 2.

**Table 2:** Mean and SD of all measurements

	Mean ± SD	Range
SF-12 (Mental health component summary score)	57.98 ± 12.54	19–100
SF-12 (Physical health component summary score)	57.67 ± 13.77	17–100
SF-12 (Total-General Health Perception)	58.64 ± 13.65	15–100
Fear of falling (VAS) (cm)	3.60 ± 3.13	0–10
Balance (BBS)	52.54 ± 3.50	35–56
Functional mobility (TUG) (sec)	13.70 ± 5.94	6–63
Proprioception (joint position sense) (°)	4.37 ± 2.84	0.2–15.2
Muscle strength (kg) (Back/leg dynomometer)	47.98 ± 22.91	20–112
Flexibility (sit and reach) (cm)	10.57 ± 13.61	-35–45

When the correlation between age, flexibility, proprioception and SF-12 was evaluated, it was observed that there was no change in quality of life with aging, flexibility and proprioception (p > 0.05).

It was found that as the body mass index (BMI) increases the quality of life decreases. Physical Health Component, Mental Health Components of SF-12 and General Health Perception of SF-12 showed the same results for BMI.

The correlation analyses between fear of falling and SF-12 showed that as the fear of falling increases, the quality of life (with the exception of the mental health component) decreases.

A strong positive correlation was observed between Physical Health Component of SF-12, General Health Perception and results of BBS. The increase in quality of life related to the increase in the balance score.

When the correlation of TUG with SF-12 was evaluated, it was found that there was a strong negative correlation between Physical Health Component of SF-12, General Health Perception and TUG. On the other hand, quality of life increased with improving functional mobility.

The analyses showed that quality of life improved as muscle strength increased since muscle strength was correlated with Physical Health Component of SF-12 and General Health Perception.

Correlation coefficients (r) and levels of significance (p) between risk factors for falls and SF-12 were given in Table 3.

**Table 3:** Pearson correlation coefficients (r) and levels of significance in a comparison of risk factors for falls and SF-12.

	SF-12 Mental Health Component	SF-12 Physical Health Component	SF-12 General Health Perception
Age	NS	NS	NS
Body Mass Index	r = -0.213 p = 0.021*	r = -0.262 p = 0.004*	r = -0.272 p = 0.003*
Fear of falling (VAS)	NS	r = -0.248 p = 0.007*	r = -0.223 p = 0.016*
Balance (BBS)	NS	r = 0.381 p = 0.000*	r = 0.270 p = 0.003*
Functional mobility (TUG)	NS	r = -0.354 p = 0.000*	r = -0.249 p = 0.007*
Proprioception (Joint position sense)	NS	NS	NS
Muscle strength (Back/leg dynamometer)	NS	r = 0.338 p = 0.000*	r = 0.230 p = 0.016*
Flexibility (Sit and reach test)	NS	NS	NS

NS: Not significant

## Discussion

In our study, the relationship between risk factors for falls and quality of life was investigated in older adults. Balance, functional mobility, muscle strength and fear of falling were shown to correlate with General Health Perception (SF-12) but no correlation was seen between proprioception and flexibility in relation to the General Health Survey (SF-12).

In the study, it was found that quality of life was not correlated with age. This result suggested that the quality of life does not change with aging but age affects the risk factors for falls.

While BMI increased, physical, mental and general health perception scores of SF-12 decreased. This relationship is important because increased BMI causes functional limitation and affects physical, mental and general health perception in older adults. In addition, the mental health component only correlated with BMI.

There are many studies which state that BBS and TUG test results are the most important risk factors for falls [10-12,14,15]. Physical and general health perception scores of SF-12 strongly correlated with BBS (positively) and TUG test (negatively). These results demonstrated that poor balance and functional mobility were associated with a decreased quality of life.

A lack of flexibility is associated with problems in executing and sustaining motor activities in daily life and is related to an increased risk of falling in older adults [19]. Proprioception is an important component of balance. Interestingly, it was seen that proprioception and flexibility did not correlated with quality of life in our study. There is a need for some further study concerning the relationship between the risk of falling, proprioception and flexibility.

When older adults worry about falling, it may indicate that their physical condition is affected, possibly due to a lack of balance. Therefore, the fear of falling is an important risk factor for falls in older adults. Hence VAS is a simple, practical and easy method of assessment for this subject and so this method was preferred. Several studies have explored the strong association between muscle strength and the risk of falling in older adults [7,8,13]. Similarly in this study, it was found that muscle strength and factors relating to the risk of falling correlated with physical and general health perception scores of SF-12.

Fear of falling correlated with Physical Health Component and General Health Perception of SF-12. Suzuki stated that fear of falling is increasingly recognized as a factor that may affect activity, function and physical condition in older adults [4].

In general while risk factors for falls were associated with physical component and general health perception of SF-12, only the body mass index correlated with the mental component of SF-12 in this study. This result pointed out that risk factors for falls affect the physical health component and general health perception but not the mental health component in older adults.

In our study balance, muscle strength, proprioception, flexibility, functional mobility were evaluated as parameters of physical function. Some investigators use physical component score of SF-12 rather than physical function. But proprioception and flexibility were not correlated with physical component score of SF-12 while muscle strength, functional mobility and balance were correlated in present study. As it is seen in our study some physical function parameters may or may not related to physical component score of quality of life.

As this study is cross sectional, the associations that were demonstrated may or may not be causal. Various factors can affect the physical or mental component scores and general quality of life in older adults. Assessing correlations only between some risk factors for falling and quality of life is the major limitation of this study.

Older adults who live in the Narlıdere Nursing Home are independent in their daily living activities. Only food and room cleaning services are provided for them. They do not spend all of their time in their own rooms but take part in activities such as visiting relatives, shopping, short holidays, taking walks etc. For this reason, some of them did not wish to participate in this study and some of them could not be reached. As a result of this, the population of this study decreased from 404 subjects to 116.

Prevention of falls and their subsequent injuries is an important goal of geriatric evaluation. Proper physical therapy programs minimizing the risk of falls may increase quality of life in older adults.

## Conclusion

The risk factors for falls (balance, functional mobility, muscle strength, fear of falling) in older adults are associated with quality of life while flexibility and proprioception are not. Future studies should focus on other factors that affect quality of life in larger elderly populations and investigate the effect of such programs on quality of life in relation to risk factors for falls.

## Competing interests

The author(s) declare that they have no competing interests.

## Authors' contributions

OA conceived of the the study and participated in the sequence alignment and drafted the study. DH carried out acquisition of data and desing of the study. GN performed the statistical analysis and drafted the manuscript. OM performed the statistical analysis. KD participated in the desing of the study. All authors read and approved the final manuscript.

## Pre-publication history

The pre-publication history for this paper can be accessed here:



## References

[B1] Dijkers M (1999). Measuring quality of life: Methodological issues. Am J Phys Med Rehabil.

[B2] Berglung AL, Ericsson K (2003). Different meanings of quality of life: a comparison between what elderly persons and geriatric staff believe is of importance. Int J Nurs Practice.

[B3] Tseng SZ, Wang RH (2001). Quality of life and related factors among elderly nursing home residents in Southern Taiwan. Public Health Nurs.

[B4] Suzuki M, Ohyama N, Yamada K, Kanamari M (2002). The relationship between fear of falling, activities of daily living and quality of life among elderly individuals. Nurs Health Sci.

[B5] Baker PS, Bodner EV, Allman RM (2003). Measuring life-space mobility in community-dwelling older adults. J Am Geriat Soc.

[B6] Downton JH (1993). Why do old people fall? Falls in the elderly.

[B7] Ringsberg K, Gerdhem P, Johansson J, Obrant KJ (1999). Is there a relationship between balance, gait performance and muscular strength in 75-year-old women?. Age Aging.

[B8] Wolfson L, Judge J, Whipple R, King M (1995). Strength is major factor in balance, gait, and the occurance of falls. J Gerontol A Biol Sci Med Sci.

[B9] Maki BE, Holliday PJ, Topper AK (1994). A prospective study of postural balance and risk of falling in a ambulatory and independent elderly population. J Gerontol.

[B10] Hatch J, Gill-Body KM, Portney LG (2003). Determinants of balance confidence in community-dwelling elderly people. Phys Ther.

[B11] Shumway-Cook A, Baldwin M, Polissar NL, Gruber W (1997). Predicting the probability for falls in community older adults. Phys Ther.

[B12] Bogle Thorbahn LD, Newton RA (1996). Use of the Berg Balance test to predict falls in elderly persons. Phys Ther.

[B13] Gehlsen GM, Whaley MH (1990). Falls in elderly: Part II, balance, strength, and flexibility. Arch Phys Med Rehabil.

[B14] Ware JE, Kosinski M, Keller SD (1996). A twelve-item short-form survey: construction of scales and preliminary tests of reliability and validity. Med Care.

[B15] Berg KO, Wood Dauphniee SL, Williams JI, Maki B (1992). Measuring balance in the elderly: validation of an instrument. Can J Public Health.

[B16] Podsiadlo D, Richardson S (1991). The timed "Up &Go": a test of basic functional mobility for frail elderly persons. J Am Geriart Soc.

[B17] Lord SR, Menz HB, Tiedemann A (2003). A Physiological profile approach to falls risk assessment and prevention. Phys Ther.

[B18] Friedman SM, Munoz B, West SK, Rubin G, Fried LP (2002). Falls and fear of falling: which comes first? A longitudinal prediction model suggests strategies for primary and secondary prevention. J Am Geriat Soc.

[B19] Lemmink KAPM, Kemper HCG, de Greef MHG, Rispens P, Stevens M (2003). The validity of the sit-and-reach test and the modified sit-and-reach test in middle-aged to older men and women. Res Q Exerc Sport.

[B20] Jones JC, Rikli RE, Max J, Noffal G (1998). The reliability and validity of a chair sit-and-reach test as a measure of hamistring flexibility in older adults. Res Q Exerc Sport.

[B21] Wolf B, Feys H, Weerdt WD, Van der Meer J, Noom M, Aufdemkampe G (2001). Effect of a physical therapeutic intervention for balance problems in the elderly: a single-blind, randomized, controlled multicentre trial. Clin Rehabil.

